# Supplementary material for: Trapping of spermine, Kukoamine A, and polyamine toxin blockers in GluK2 kainate receptor channels
Source: Nat Commun. 2024 Nov 26;15:10257. doi: 10.1038/s41467-024-54538-x (PMC11599716; doi:10.1038/s41467-024-54538-x)
Supplement: Supplementary file 1 — Supplementary Information [file 41467_2024_54538_MOESM1_ESM.pdf]

Supplementary Information

Trapping of spermine, Kukoamine A, and polyamine toxin blockers in GluK2 kainate receptor channels

Shanti Pal Gangwar[1,#], Maria V. Yelshanskaya[1,#], Muhammed Aktolun[2,#], Laura Y. Yen[1,3], Thomas P. Newton[1,4], Kristian Strømgaard[5], Maria G. Kurnikova[2] and Alexander I. Sobolevsky[1*]

[1] Department of Biochemistry and Molecular Biophysics, Columbia University, 650 West 168th Street, New York, NY 10032, USA

[2] Department of Chemistry, Carnegie Mellon University, Pittsburgh, PA 15213, USA

[3] Cellular and Molecular Physiology and Biophysics Graduate Program, Columbia University Irving Medical Center, 630 West 168th Street, New York, NY 10032, USA

[4] Integrated Program in Cellular, Molecular and Biomedical Studies, Columbia University Irving Medical Center, 630 West 168th Street, New York, NY 10032, USA

[5] Center for Biopharmaceuticals, Department of Drug Design and Pharmacology, University of Copenhagen, Jagtvej 162, DK-2100 Copenhagen, Denmark

[#] These authors contributed equally to this work

[*] Correspondence and requests for materials should be addressed to A.I.S. (Email: as4005@cumc.columbia.edu; Tel: 212-305-4249)

This PDF file includes:

Supplementary Figures 1-7

Supplementary Tables 1-3

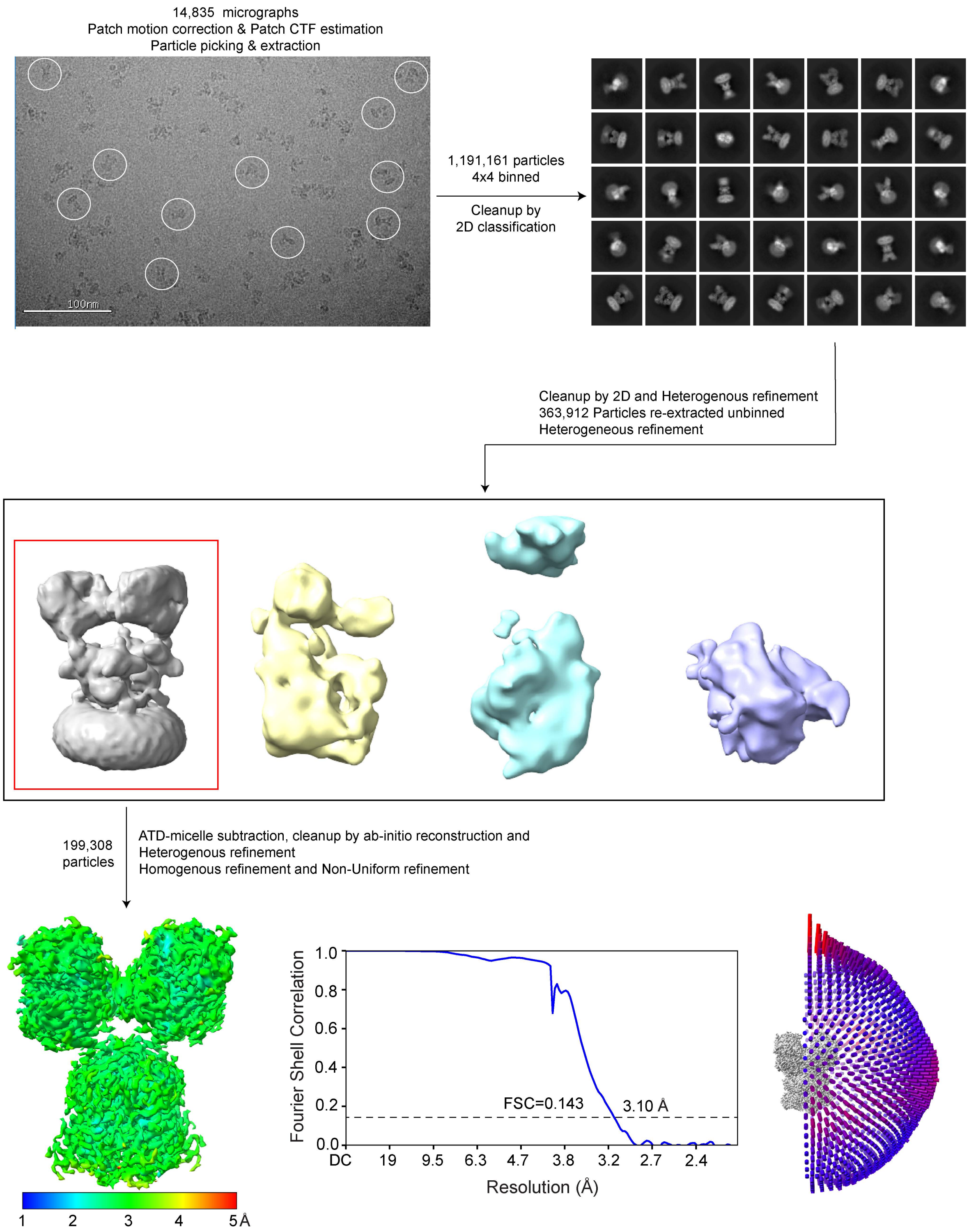

Supplementary Fig. 1. Overview of cryo-EM for GluK2$_{\mathbf{NpTx8}}$. On the top, 3D reconstruction workflow, with a representative micrograph and 2D class averages. At the bottom, the local resolution presented as coloring of the GluK2$_{NpTx8}$ map, FSC curve, and Euler angle distribution of particles contributing to the final reconstruction, with larger red cylinders representing orientations comprising more particles.

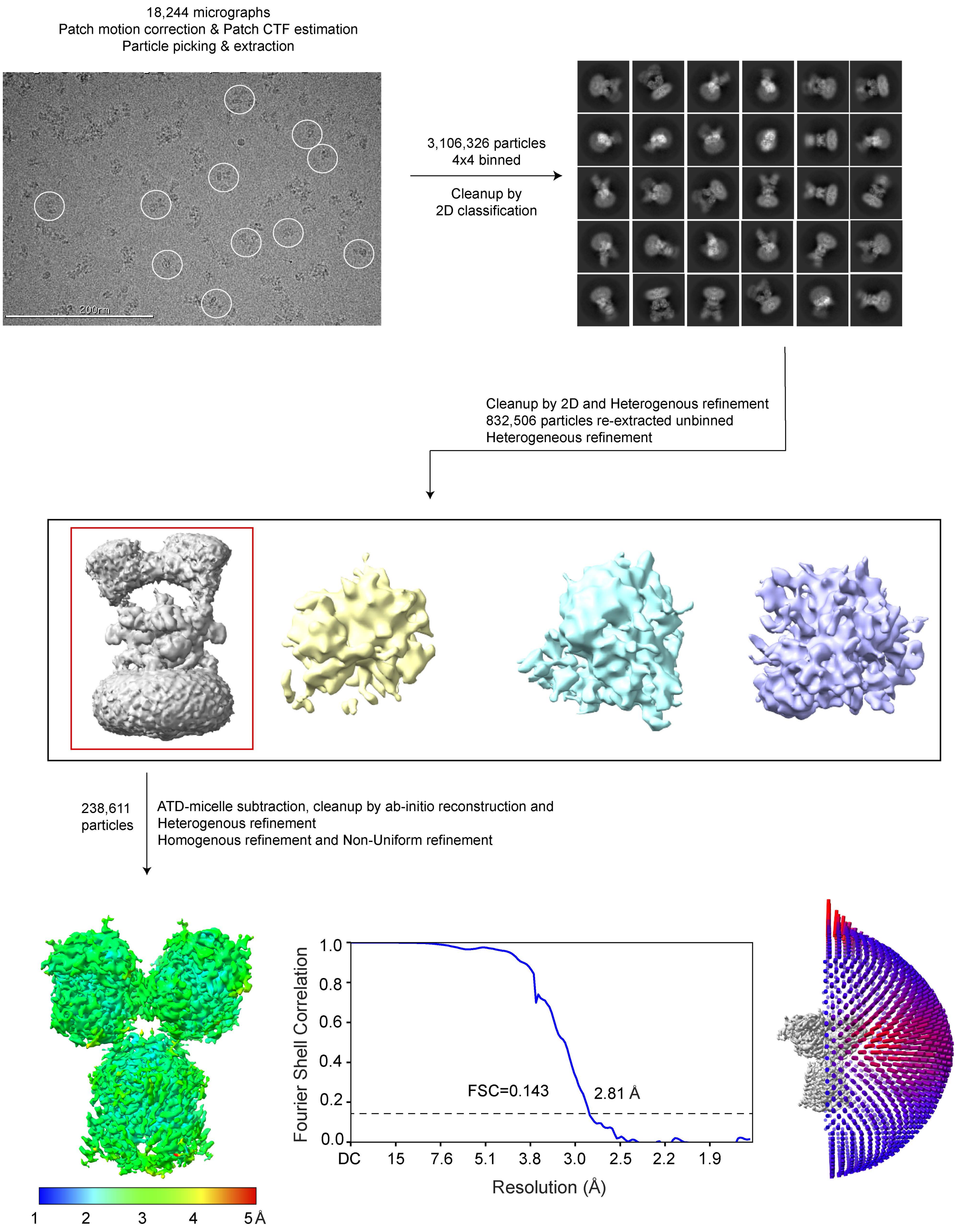

Supplementary Fig. 2. Overview of cryo-EM for GluK2$_{PhTx74}$. On the top, 3D reconstruction workflow, with a representative micrograph and 2D class averages. At the bottom, the local resolution presented as coloring of the GluK2$_{PhTx74}$ map, FSC curve, and Euler angle distribution of particles contributing to the final reconstruction, with larger red cylinders representing orientations comprising more particles.

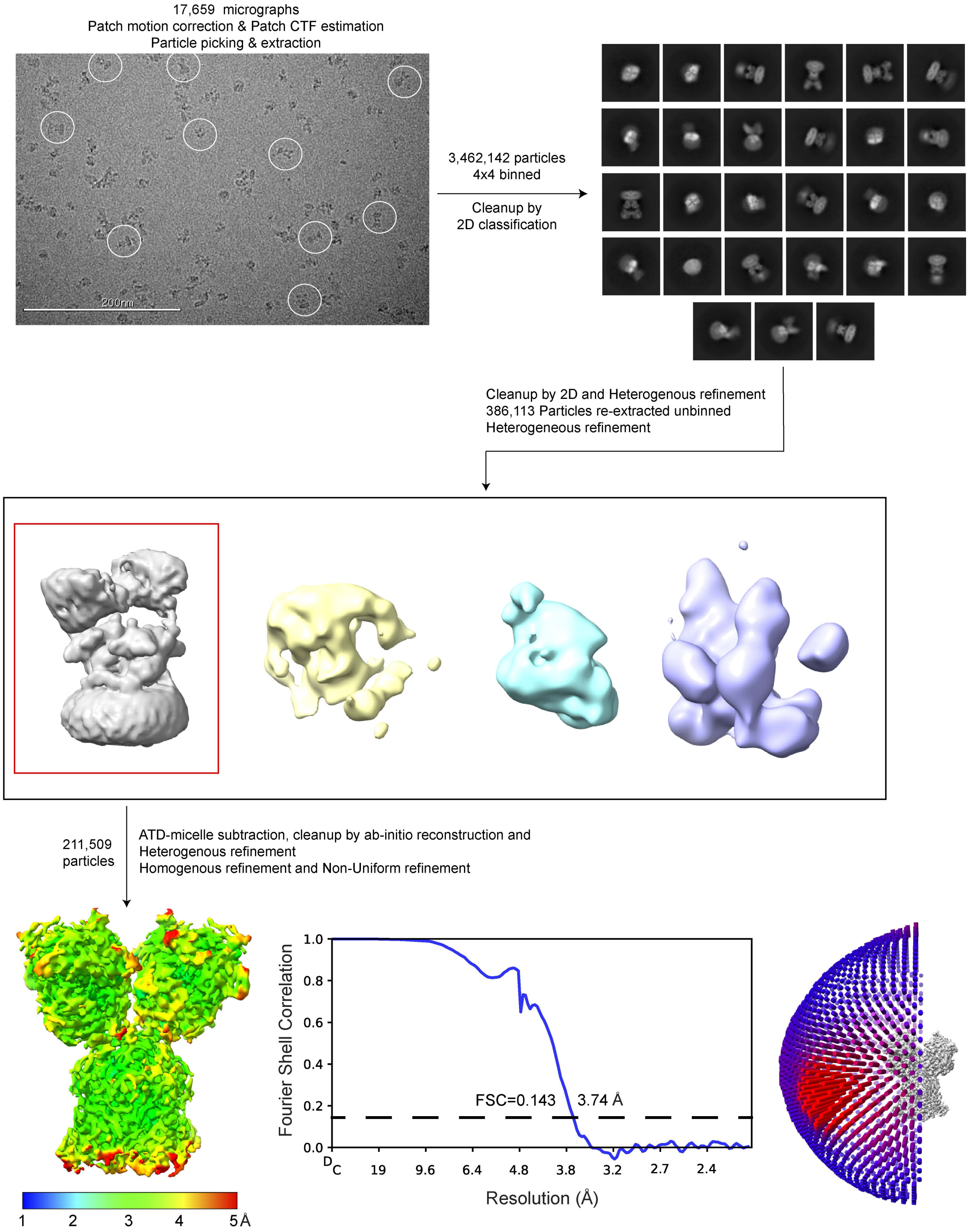

Supplementary Fig. 3. Overview of cryo-EM for GluK2$_{KukoA}$. On the top, 3D reconstruction workflow, with a representative micrograph and 2D class averages. At the bottom, the local resolution presented as coloring of the GluK2$_{KukoA}$ map, FSC curve, and Euler angle distribution of particles contributing to the final reconstruction, with larger red cylinders representing orientations comprising more particles.

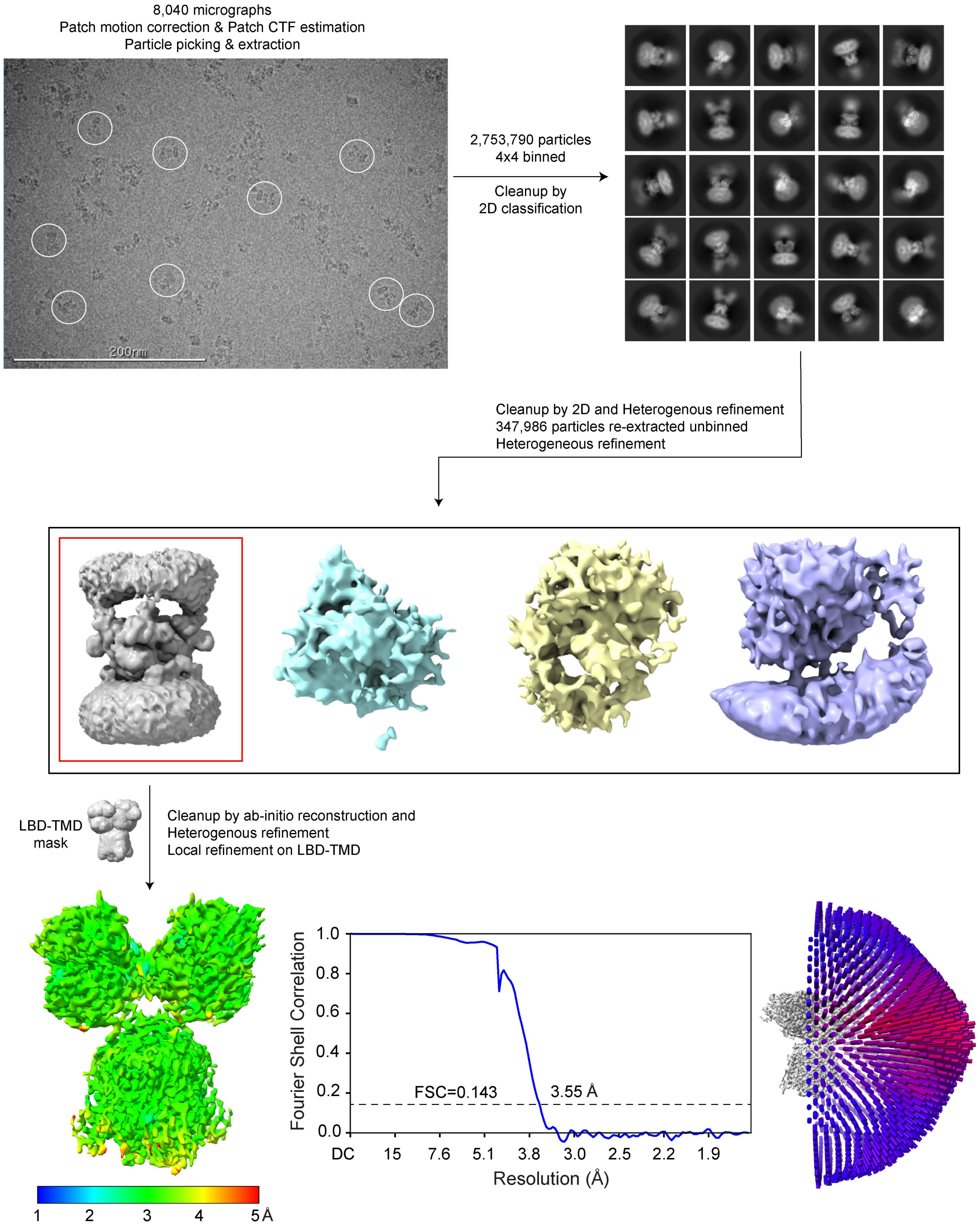

Supplementary Fig. 4. Overview of cryo-EM for GluK2$_{SPM}$. On the top, 3D reconstruction workflow, with a representative micrograph and 2D class averages. At the bottom, the local resolution presented as coloring of the GluK2$_{SPM}$ map, FSC curve, and Euler angle distribution of particles contributing to the final reconstruction, with larger red cylinders representing orientations comprising more particles.

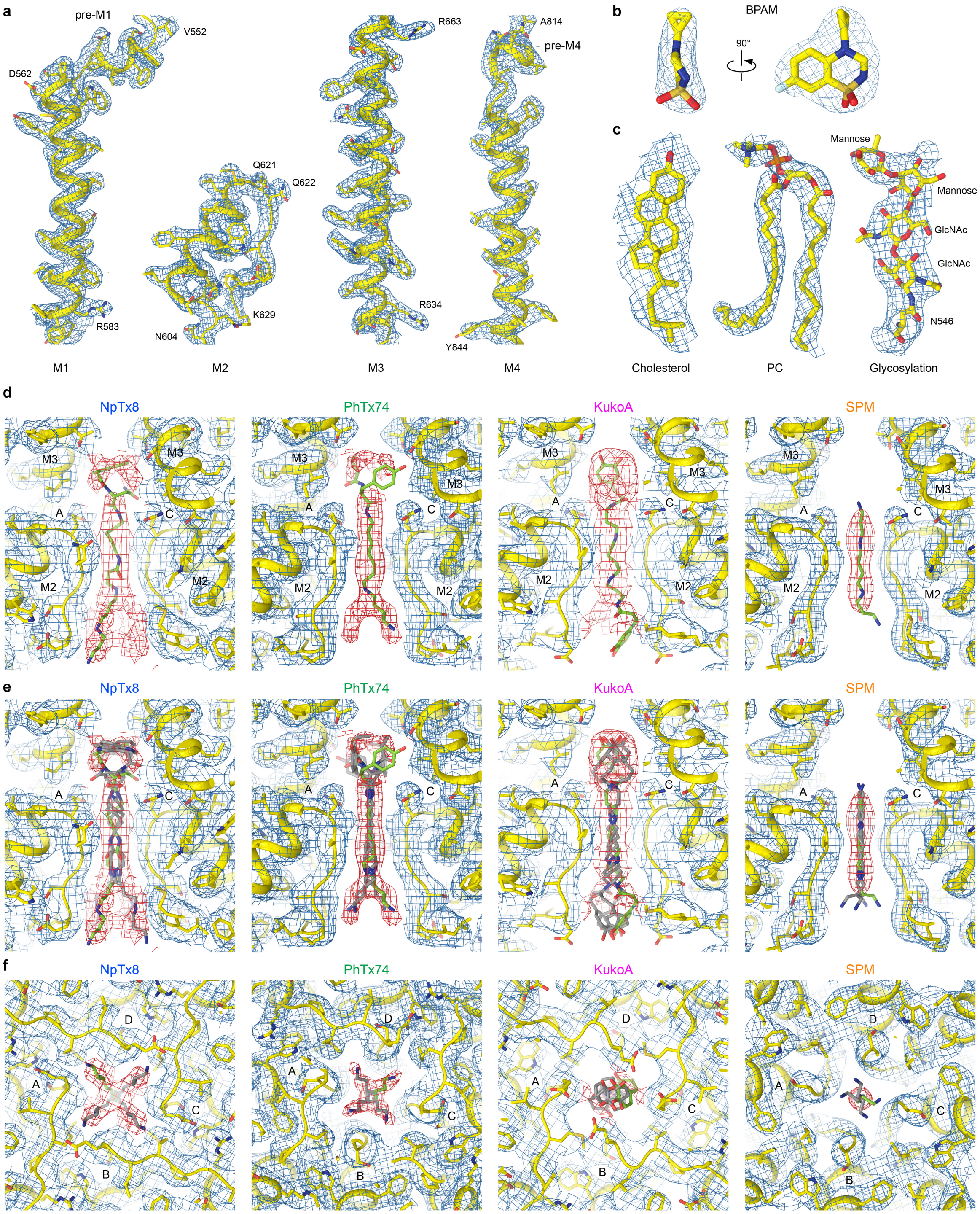

Supplementary Fig. 5. Cryo-EM density. a, Fragments of the GluK2$_{PhTx74}$ TMD with the struc:ural model shown as a ribbon and sticks and the corresponding cryo-EM density as a blue mesh. **b-c,** Representative GluK2$_{PhTx74}$ density for the positive allosteric modulator BPAM (**b**), lipids cholesterol and phosphatidylcholine (PC), and N546-linked carbohydrate (**c**), with the molecular models shown in sticks (yellow). **d-e,** Closeup view of the blocker binding site in GluK2$_{NpTx8}$, GluK2$_{PhTx74}$, GluK2$_{KukoA}$ and GluK2$_{SPM}$, with density for the channel blockers and the surrounding protein shown as red and blue mesh, respectively. The protein model is shown in yellow. One fitted blocker

molecule (**d**) is shown in green, while its three equivalent poses obtained via 90-degree rotations around the axis of local 4-fold rotational symmetry (**e**) are shown in grey. Only two subunits (A and C) are shown for the protein, with the other two subunits (B and D) removed for clarity. **f,** Intracellular view of the same region as shown in **e** but with all four protein subunits present.

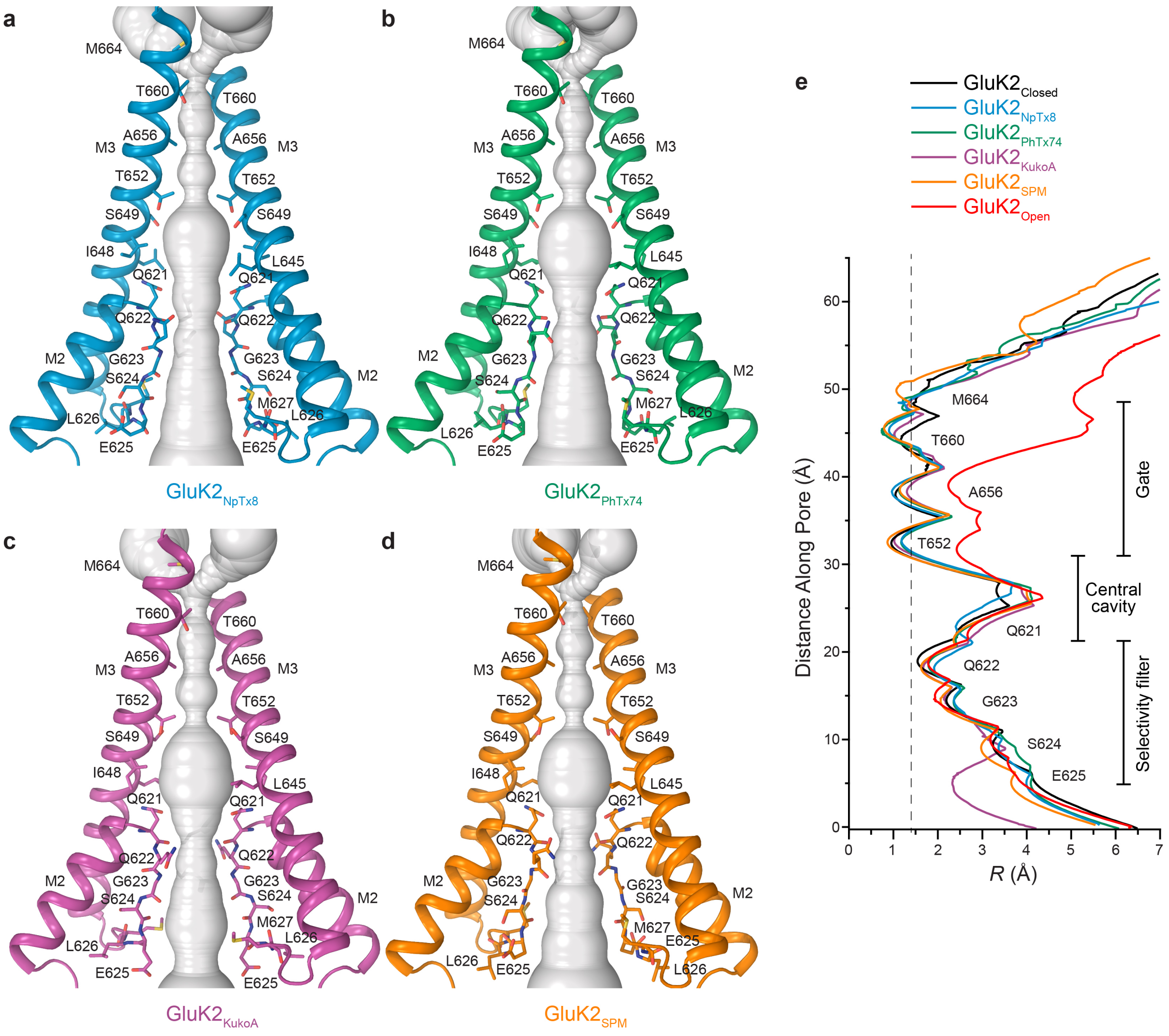

Supplementary Fig. 6. Closed conformation of the ion channel pore. a-c, Pore-forming domains M2 and M3 in GluK2$_{NpTx8}$ (**a**), GluK2$_{PhTx74}$ (**b**), GluK2$_{KukoA}$ (**c**) and GluK2$_{SPM}$ (**d**), with the residues lining pore shown as sticks. Only two (A and C) of four subunits are shown, with the front and back subunits (B and D) omitted for clarity. The pore profile is shown as a space-filling model (grey). **e**, Pore radius for GluK2$_{Closed}$ (black; PDB ID: 8FWS), GluK2$_{NpTx8}$ (blue), GluK2$_{PhTx74}$ (green), GluK2$_{KukoA}$ (violet), GluK2$_{SPM}$ (orange) and GluK2$_{Open}$ (red; PDB ID: 9B35) calculated using HOLE. The vertical dashed line denotes the radius of a water molecule, 1.4 Å.

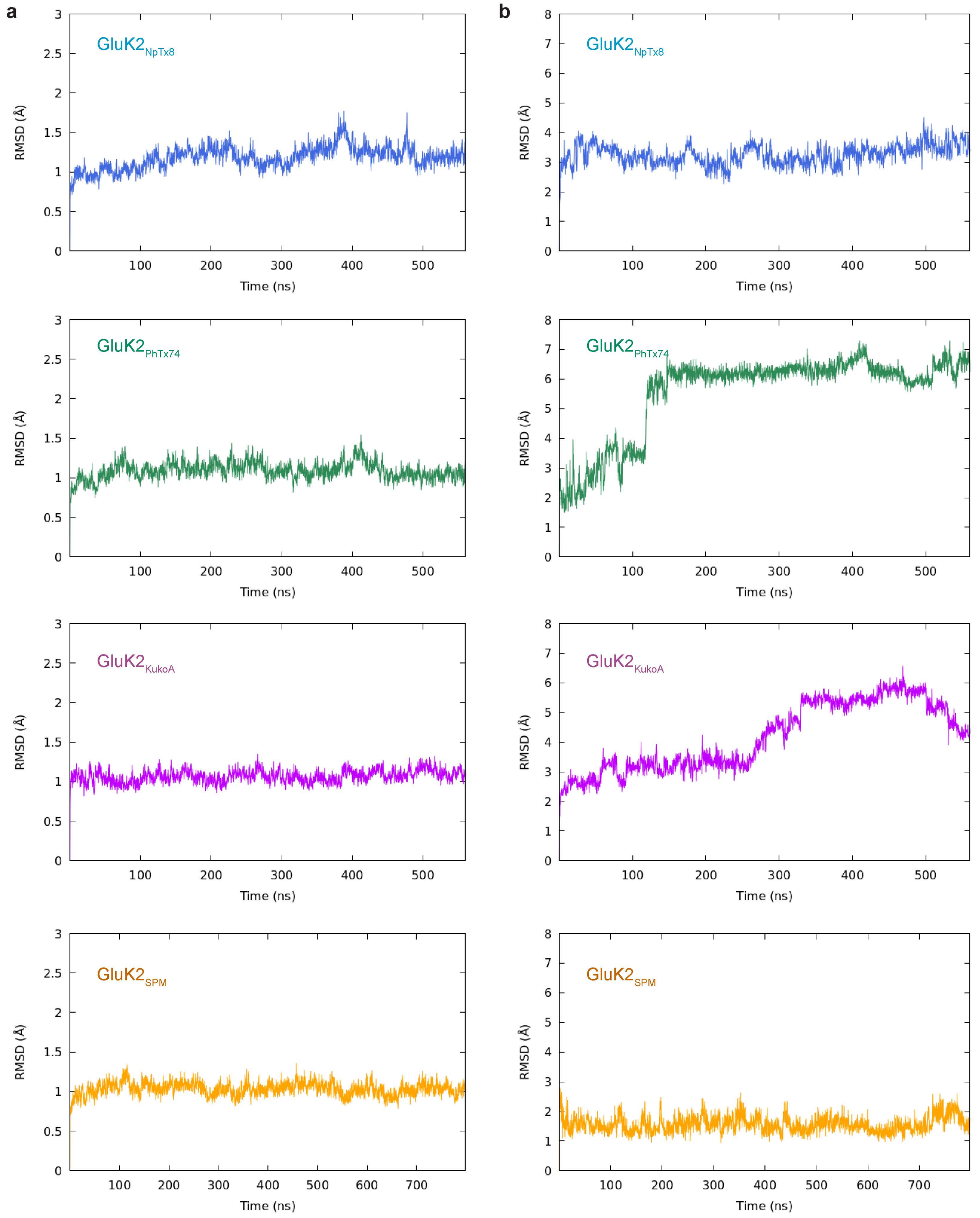

Supplementary Fig. 7. Protein and blocker stability during MD simulations. a, Root-mean square deviation (RMSD) from the initial structures in the production runs for GluK2$_{NpTx8}$ (blue), GluK2$_{PhTx74}$ (green), GluK2$_{KukoA}$ (violet) and GluK2$_{SPM}$ (orange) systems. For each system, Cα RMSDs of the M1-4 TMD segments are shown. **b**, RMSDs of the blocker molecules NpTx-8 (blue), PhTx-74 (green), KukoA (violet) and SPM (orange), from the initial conformations in the production runs of corresponding systems. The alignment was done by the TMD helices and the heavy-atom RMSDs of the blockers in each system are shown.

Supplementary Table 1. Heavy-atom contact frequencies between the channel blockers and key protein residues during the simulations. Contacts that lasted for less than 60% of the simulation are not shown.

Simulation System	GluK2 Subunit	Contact Frequency (%)									
		Q621	Q622	G623	S624	E625	M627	L645	I648	S649	T652
$GluK2_{SPM}$	A	99.70	98.07	-	62.14	89.16	-	-	-	-	-
	B	98.67	90.19	-	-	-	-	-	-	-	-
	C	98.18	84.63	-	-	-	-	-	-	-	-
	D	91.83	88.22	-	62.56	69.22	-	-	-	-	-
$GluK2_{KukoA}$-1	A	95.72	91.08	-	60.10	-	-	-	-	70.45	-
	B	97.36	-	-	-	-	-	-	63.05	-	89.20
	C	100.00	78.81	-	-	62.73	-	-	-	-	-
	D	99.84	96.72	-	72.93	72.41	67.37	-	60.66	-	-
$GluK2_{KukoA}$-2	A	100.00	96.81	-	80.07	-	-	-	84.95	-	-
	B	95.38	-	-	-	-	-	-	-	-	-
	C	98.19	98.38	-	-	-	-	-	75.64	-	87.13
	D	99.81	73.33	97.75	100.00	-	99.94	83.76	68.96	-	79.89
$GluK2_{NpTx8}$	A	84.36	-	-	-	-	-	82.50	65.52	-	-
	B	96.54	90.36	-	-	-	-	-	-	-	-
	C	100.00	98.34	94.82	-	-	-	-	83.84	94.77	60.70
	D	97.84	99.80	-	-	-	-	-	-	-	83.04
$GluK2_{PhTx74}$	A	79.95	-	-	-	-	-	61.45	73.57	92.25	78.82
	B	99.95	63.86	-	-	-	-	66.30	95.07	-	98.20
	C	99.11	-	-	-	-	-	-	69.45	80.27	99.73
	D	98.93	-	-	-	-	-	-	-	-	74.75

Supplementary Table 2. List of hydrogen bond interactions between the channel blockers and the binding site residues. Duty fraction refers to the percentage of frames in which a given hydrogen bond is present in the trajectory. Hydrogen bonds that persist less than 20% of the trajectory are not listed. Single asterisk (*) indicates the alternating hydrogen bond atoms within the same residue. Double asterisk (**) indicates the alternating hydrogen bonds among the subunits.

Simulation System	GluK2 Subunit	Hydrogen bonds (Acceptor-Donor)	Duty Fraction (%)
GluK2$_{SPM}$	A	**Q621:O-----SPM:N5	89.38
		**Q622:O-----SPM:N10	47.93
		*E625:OE1/2-----SPM:N14	100.00
		**Q621:OE1-----SPM:N1	57.27
	B	**Q621:O-----SPM:N5	67.77
		**Q621:OE1-----SPM:N1	39.58
		*E625:OE1/2-----SPM:N14	26.02
	C	**Q621:O-----SPM:N5	23.73
		*E625:OE1/2-----SPM:N14	31.85
		**Q621:OE1-----SPM:N1	21.3
		**Q622:O-----SPM:N10	25.70
	D	**Q621:O-----SPM:N5	44.97
		*E625:OE1/2-----SPM:N14	100.00
		**Q622:O-----SPM:N10	44.32
		**Q621:OE1-----SPM:N1	34.09
		S624:O-----SPM:N14	22.49
GluK2$_{KukoA}$-1	A	**Q622:O-----KUK:N15	52.2
		Q621:OE1-----KUK:N24	45.04
		**Q621:O-----KUK:N20	38.90
	B	T652:OG1-----KUK:O35	74.00
		**Q621:O-----KUK:N20	20.01
	C	**Q621:O-----KUK:N20	72.44
		KUK:O26-----Q621:NE2	95.40
		**Q622:O-----KUK:N15	43.11
	D	**Q621:O-----KUK:N20	83.36
		**Q622:O-----KUK:N15	64.04
GluK2$_{KukoA}$-2	A	Q622:O-----KUK:O37	93.38
		*Q621:OE1/NE2-----KUK:O36	99.07
	B	**Q622:O-----KUK:N11	35.35
	C	**Q621:O-----KUK:N15	34.54
		KUK:O26-----T652:OG1	66.46
		KUK:O10-----S624:N	31.54
		**Q621:OE1-----KUK:N20	40.23
	D	**Q622:O-----KUK:N11	56.53
		**Q621:O-----KUK:N15	59.90
		*Q621:OE1/NE2-----KUK:O35	35.11
		**Q621:OE1-----KUK:N20	50.47
		*KUK:O35/36-----Q621:NE2	98.87
		*KUK:O37/38-----S624:N	50.66
GluK2$_{NpTx8}$	A	Q621:OE1-----NTX:N6	63.28
		S624:O-----NTX:N2	26.65
		*E625:OE1/2-----NTX:N1	56.25
		Q622:OE1-----NTX:N3	29.90
		Q621:O-----NTX:N4	61.17
	B	**Q622:O-----NTX:N3	63.24
		*E625:OE1/2-----NTX:N1	53.93
		NTX:O4-----Q621:NE2	53.02
		NTX:O3-----T652:OG1	23.09
	C	S649:OG-----NTX:N8	56.23
		Q621:OE1-----NTX:N8	37.38
	D	*Q622:OE1/NE2-----NTX:N4	97.73
		**Q622:O-----NTX:N3	25.38
GluK2$_{PhTx74}$	A	**Q622:O-----PTX:N4	56.02
		PTX:O1-----T652:OG1	22.94
	B	Q621:OE1/NE2-----PTX:O2	100.00
		**Q622:O-----PTX:N4	37.36
		Q621:O-----PTX:N3	20.43
		PTX:O1-----T652:OG1	81.84
		PTX:O2-----Q621:NE2	36.64
	C	**Q621:O-----PTX:N3	22.82
		PTX:O3-----T652:OG1	57.02
		**Q622:O-----PTX:N4	22.13
	D	**Q621:O-----PTX:N3	78.68

Supplementary Table 3. List of hydrophobic interactions between the channel blockers and the hydrophobic side chains of the binding site residues. Duty fraction refers to the percentage of frames in which a given hydrophobic contact is present in the trajectory. Contacts that persist less than 20% of the trajectory are not listed.

Simulation System	GluK2 Subunit	Hydrophobic Contacts	Duty Fraction (%)
**GluK2$_{SPM}$**	A	-	-
	B	-	-
	C	-	-
	D	-	-
**GluK2$_{KukoA}$-1**	A	KUK:C34-----I648:CG2	29.70
	B	-	-
	C	KUK:C28-----I648:CG2	33.10
	D	KUK:C27-----I648:CD1	47.00
**GluK2$_{KukoA}$-2**	A	KUK:C31-----I648:CG2	36.50
		KUK:C30-----I648:CG2	30.90
		KUK:C32-----I648:CG2	25.70
		KUK:C30-----T652:CG2	20.10
	B	-	-
	C	KUK:C23-----I648:CG1	34.80
		KUK:C23-----I648:CG2	25.90
	D	KUK:C4-----M627:CE	97.40
		KUK:C3-----M627:CE	91.40
		KUK:C2-----M627:CG	81.60
		KUK:C3-----M627:CG	81.10
		KUK:C5-----M627:CE	80.00
		KUK:C2-----M627:CE	49.90
		KUK:C6-----M627:CE	38.10
		KUK:C1-----M627:CE	24.70
		KUK:C3-----G623:C	23.30
		KUK:C33-----L645:CD1	23.00
		KUK:C33-----I648:C	22.20
**GluK2$_{NpTx8}$**	A	NTX:C18-----L645:CD1	47.40
		NTX:C19-----L645:CD1	29.20
		NTX:C18-----I648:CG2	35.40
	B	NTX:C21-----L645:CD1	33.60
		NTX:C20-----I648:CG2	29.80
	C	NTX:C23-----I648:CG2	61.10
		NTX:C22-----I648:CG2	34.40
		NTX:C24-----I648:CG2	27.90
		NTX:C21-----I648:CG2	22.70
		NTX:C28-----T652:CG2	25.50
	D	-	-
**GluK2$_{PhTx74}$**	A	PTX:C1-----L645:CD1	43.00
		PTX:C3-----I648:CG2	31.80
		PTX:C1-----I648:CG2	23.90
		NTX:C4-----T652:CG2	20.20
	B	PTX:C12-----I648:CG2	72.80
		PTX:C11-----I648:CG2	65.70
		PTX:C7-----I648:CG2	44.00
		PTX:C9-----L645:CD1	35.90
		PTX:C12-----I648:CG1	32.70
		PTX:C10-----I648:CG2	32.00
		PTX:C9-----I648:CG2	24.80
		PTX:C8-----I648:CG2	24.20
	C	PTX:C9-----I648:CG2	47.10
		PTX:C15-----L645:CD1	27.60
		PTX:C17-----L645:CD1	27.20
	D	-	-
