# Supplementary material for: Trapping of spermine, Kukoamine A, and polyamine toxin blockers in GluK2 kainate receptor channels
Source: Nat Commun. 2024 Nov 26;15:10257. doi: 10.1038/s41467-024-54538-x (PMC11599716; doi:10.1038/s41467-024-54538-x)
Supplement: Supplementary file 2 — Reporting Summary [file 41467_2024_54538_MOESM2_ESM.pdf]

nature portfolio

Corresponding author(s): Alexander I. Sobolevsky

Last updated by author(s): Oct 29, 2024

Reporting Summary

Nature Portfolio wishes to improve the reproducibility of the work that we publish. This form provides structure for consistency and transparency in reporting. For further information on Nature Portfolio policies, see our Editorial Policies and the Editorial Policy Checklist.

Statistics

For all statistical analyses, confirm that the following items are present in the figure legend, table legend, main text, or Methods section.

n/a	Confirmed	
☐	☒	The exact sample size (*n*) for each experimental group/condition, given as a discrete number and unit of measurement
☐	☒	A statement on whether measurements were taken from distinct samples or whether the same sample was measured repeatedly
☐	☒	The statistical test(s) used AND whether they are one- or two-sided<br>*Only common tests should be described solely by name; describe more complex techniques in the Methods section.*
☒	☐	A description of all covariates tested
☒	☐	A description of any assumptions or corrections, such as tests of normality and adjustment for multiple comparisons
☐	☒	A full description of the statistical parameters including central tendency (e.g. means) or other basic estimates (e.g. regression coefficient) AND variation (e.g. standard deviation) or associated estimates of uncertainty (e.g. confidence intervals)
☐	☒	For null hypothesis testing, the test statistic (e.g. *F*, *t*, *r*) with confidence intervals, effect sizes, degrees of freedom and *P* value noted<br>*Give P values as exact values whenever suitable.*
☒	☐	For Bayesian analysis, information on the choice of priors and Markov chain Monte Carlo settings
☒	☐	For hierarchical and complex designs, identification of the appropriate level for tests and full reporting of outcomes
☒	☐	Estimates of effect sizes (e.g. Cohen's *d*, Pearson's *r*), indicating how they were calculated

Our web collection on statistics for biologists contains articles on many of the points above.

Software and code

Policy information about availability of computer code

Data collection	Legion 3.5, pCLAMP 10.2
Data analysis	cryoSPARC 4.4.1, UCSF Chimera 1.16, COOT 0.9.8.1, PHENIX 1.18, pyMOL 2.5.2, HOLE 2.1, Origin 2023, pCLAMP 10.2, CHARMM-GUI, AmberTools20, Amber20, VMD 1.9.4

For manuscripts utilizing custom algorithms or software that are central to the research but not yet described in published literature, software must be made available to editors and reviewers. We strongly encourage code deposition in a community repository (e.g. GitHub). See the Nature Portfolio guidelines for submitting code & software for further information.

Data

Policy information about availability of data

All manuscripts must include a data availability statement. This statement should provide the following information, where applicable:

- Accession codes, unique identifiers, or web links for publicly available datasets
- A description of any restrictions on data availability
- For clinical datasets or third party data, please ensure that the statement adheres to our policy

All data needed to evaluate the conclusions of the paper are present in the paper. Cryo-EM density maps have been deposited to the Electron Microscopy Database (EMDB) under the accession codes EMD-47295 [https://www.ebi.ac.uk/pdbe/entry/emdb/EMD-47295] for GluK2PhTx74, EMD-47296 [https://www.ebi.ac.uk/pdbe/entry/emdb/EMD-47296] for GluK2NpTx8, EMD-47297 [https://www.ebi.ac.uk/pdbe/entry/emdb/EMD-47297] for GluK2SPM, and EMD-47298 [https://

www.ebi.ac.uk/pdbe/entry/emdb/EMD-47298] for GluK2KukoA. The corresponding atomic coordinates have been deposited to the Protein Data Bank (PDB) under the accession codes 9DXQ [https://doi.org/10.2210/pdb9DXQ/pdb] for GluK2PhTx74, 9DXR [https://doi.org/10.2210/pdb9DXR/pdb] for GluK2NpTx8, 9DXS [https://doi.org/10.2210/pdb9DXS/pdb] for GluK2SPM, and 9DXT [https://doi.org/10.2210/pdb9DXT/pdb] for GluK2KukoA. The accession codes for previously published structures that were used for model building or illustrations: 8FWS [https://doi.org/10.2210/pdb8FWS/pdb] for GluK2closed, and 9B35 [https://doi.org/10.2210/pdb9B35/pdb] for GluK2open. The molecular dynamics simulation data without lipid and water coordinates, together with topology files, have been deposited in Zenodo under accession code 10.5281/zenodo.14009849. The repository also includes the full initial coordinates (with waters and lipids) for the production runs as well as the representative structures that correspond to the structures in Fig. 6, as described in the Methods section. The raw simulation data have not been deposited due to their size, but access can be obtained by contacting the authors. The source data underlying Fig. 1c,e are provided as a Source Data file.

Research involving human participants, their data, or biological material

Policy information about studies with human participants or human data. See also policy information about sex, gender (identity/presentation), and sexual orientation and race, ethnicity and racism.

Reporting on sex and gender	N/A
Reporting on race, ethnicity, or other socially relevant groupings	N/A
Population characteristics	N/A
Recruitment	N/A
Ethics oversight	N/A

Note that full information on the approval of the study protocol must also be provided in the manuscript.

Field-specific reporting

Please select the one below that is the best fit for your research. If you are not sure, read the appropriate sections before making your selection.

☒ Life sciences ☐ Behavioural & social sciences ☐ Ecological, evolutionary & environmental sciences

For a reference copy of the document with all sections, see nature.com/documents/nr-reporting-summary-flat.pdf

Life sciences study design

All studies must disclose on these points even when the disclosure is negative.

Sample size	Amount of cryo-EM data collected was limited by time allocation at the microscopes. For electrophysiological experiments, we only selected healthy-looking cells, with contrast body and smooth membrane that also showed the fluorescent signal of GFP expressed under a different from GluK2 promotor. No statistical approaches were used to predetermine the sample size but all measurements were performed using five or more biologically independent measurements. Exact number of biologically independent measurements and the number of independent experiments are reported in the figure legends.
Data exclusions	No data has been excluded.
Replication	No replication attempts have failed. Cryo-EM data collections were performed during two continuous two- and three-day data collection sessions and were consistent from the beginning to the end. A replication of the cryo-EM data collection was therefore not necessary or economically justifiable. In electrophysiological experiments, we made at least five independent replicates for each type of experiment.
Randomization	Samples were not randomized; it is not technically or practically feasible to do so for cryo-EM or patch-clamp studies. Covariant control is not economically viable in cryo-EM data collections. Covariant control was also not possible in electrophysiological experiments due to the need to transfect with predetermind cDNAs and optimize protein expression for an individual construct.
Blinding	Researchers were not blinded; it is not technically or practically feasible to do so for cryo-EM or electrophysiological experiments. It is not economically viable to blind cryo-EM collections. For electrophysiological experiments, researchers conducting the studies were also in charge of cell as well as protein expression optimization for individual constructs in order to achieve recordings or transfected cells in these studies. These circumstances made blinding not possible.

Reporting for specific materials, systems and methods

We require information from authors about some types of materials, experimental systems and methods used in many studies. Here, indicate whether each material, system or method listed is relevant to your study. If you are not sure if a list item applies to your research, read the appropriate section before selecting a response.

Materials & experimental systems

n/a	Involved in the study
☒	☐ Antibodies
☐	☒ Eukaryotic cell lines
☒	☐ Palaeontology and archaeology
☒	☐ Animals and other organisms
☒	☐ Clinical data
☒	☐ Dual use research of concern
☒	☐ Plants

Methods

n/a	Involved in the study
☒	☐ ChIP-seq
☒	☐ Flow cytometry
☒	☐ MRI-based neuroimaging

Eukaryotic cell lines

Policy information about cell lines and Sex and Gender in Research

Cell line source(s)	HEK 293S GnTI-, ATCC, Cat#CRL-3022<br>Sf9, Gibco, Cat#12659017<br>HEK 293, ATCC, Cat#CRL-1573
Authentication	None of the cell lines used have been authenticated.
Mycoplasma contamination	The cell lines used have been tested for mycoplasma contamination by the providers (negative results) but have not been retested in the lab.
Commonly misidentified lines<br>(See ICLAC register)	No commonly misidentified lines were used in this study.

Plants

Seed stocks	N/A
Novel plant genotypes	N/A
Authentication	N/A
